# Lance-Adams Syndrome: An Updated Review of a Rare Post-Hypoxic Complication

**DOI:** 10.5334/tohm.1074

**Published:** 2025-09-24

**Authors:** Mohammad Malakooti, Afshin Heidari, Mahsa Motieian, Armita Farid, Sina Neshat, Matin Bidares, Fatemeh Jahanshahi, Milad Gorgani, Hossein Shayestehyekta, Hazhir Moradi, Aydin Valipoor

**Affiliations:** 1 Department of Neurology, Faculty of Medicine, Iran University of Medical Sciences, Tehran, Iran; 2 School of Medicine, Isfahan University of Medical Sciences, Isfahan, Iran; 3 Independent researcher, Dobbs Ferry, NY, United States; 4 Faculty of Medicine, Iran University of Medical Sciences, Tehran, Iran; 5 Department of Biostatistics and Epidemiology, San Francisco, CA, United States; 6 Health Policy Research Center, Shiraz University of Medical Sciences, Shiraz, Iran; 7 Student Research Committee, Faculty of Medicine, Iran University of Medical Sciences, Tehran, Iran; 8 Clinical and Translational Science Institute, University of Miami Miller School of Medicine, Miami, FL, United States; 9 Nosocomial Research Center, Isfahan University of Medical Sciences, Isfahan, Iran; 10 Department of Emergency Medicine, School of Medicine, Iran University of Medical Sciences, Tehran, Iran

**Keywords:** Lance-Adams Syndrome, Post-Hypoxic Myoclonus, Myoclonus, Cardiopulmonary Resuscitation

## Abstract

**Objectives:**

This review aims to provide a comprehensive understanding of Lance-Adams Syndrome (LAS), focusing on its pathophysiology, diagnosis, management, and patient outcomes.

**Methods:**

Utilizing the PICO framework, articles describing known cases of LAS and interventions were included, with no specific comparisons. The search was conducted in Google Scholar, PubMed, and Scopus databases.

**Results:**

LAS is characterized by action myoclonus and related symptoms. Imbalances in neurotransmitters and involvement of brain areas like the cerebellum, thalamus, and basal ganglia play a role in its pathophysiology. Diagnosis primarily relies on symptom history post-anoxic events, and treatments vary in effectiveness. LAS generally carries a more favorable functional prognosis than early post-anoxic status myoclonus, but often remains chronic and disabling.

**Conclusion:**

LAS is a complex and rare neurological condition requiring early diagnosis and specific interventions. The compiled information provides a comprehensive overview, assisting clinicians in understanding and managing LAS. This review emphasizes the need for ongoing research and individualized patient care strategies, seeking to enhance overall patient quality of life.

## 1. Introduction

Lance-Adams syndrome (LAS) was first described by James W. Kance and Raymond D. Adams in the 1960s. During Kance’s fellowship at Massachusetts General Hospital in Boston, the two presented four cases in a paper. The cases showed symptoms of dysarthria, ataxia, and nystagmus following hypoxia caused by cardiac arrest or airway obstruction and impaired consciousness for several days. The disorders were more severe in two cases in the upper limb and two in the lower limb. The common finding among all four patients was a degree of involuntary movements and intention tremors. These movements were referred to as *myoclonic jerks* in the study by Kance and Adams. This generalized myoclonus, especially during active and voluntary movements, was the characteristic hallmark of the condition that appeared a few days after hypoxia. The arrhythmic movements, which occurred in a muscle or group of muscles, were intensified during emotional stimulation but were suppressed by treatment with barbiturates [1].

Lance-Adams syndrome is a chronic and rare form of post-hypoxic myoclonus, a neurological condition marked by myoclonic jerks following events leading to hypoxia [2,3,4]. A specific set of clinical symptoms characterizes LAS. As a primary manifestation, action myoclonus manifests as repetitive action myoclonic jerk that may be generalized, focal, or multifocal [5]. A sensory stimulus can trigger myoclonus in some patients. A patient with LAS may experience both positive and negative subcortical or cortical myoclonus. In the case of negative myoclonus, walking posture can be impaired in the lower extremities, and falls may occur. Moreover, positive myoclonus can be characterized by jerky, brief movements of the limbs. Patients with action myoclonus experience multiple difficulties in coping with the condition, as it lasts their entire lives, impairs their function and decreases their quality of life [6].

This comprehensive review of databases is being conducted because we could not find any other recent literature review on this topic. This review is being performed to provide a more thorough understanding of the condition by compiling all information known about LAS.

## 2. Methods

### 2.1 PICO Framework

**Population:** Articles describing known cases of Lance-Adams syndrome were included.

**Intervention:** Articles describing interventions for treating and controlling Lance-Adams syndrome were included.

**Comparison:** Not applicable.

**Outcome:** All variables relevant to comparing the studies and measuring outcomes were extracted.

### 2.2 Search Strategy

A search for relevant articles has been conducted using key terms such as “lance-adams” [Title/Abstract], “chronic post-hypoxic myoclonus” [Title/Abstract]), and using “Myoclonus” [Mesh] combined with “Heart Arrest” [Mesh] OR “Cardiopulmonary Resuscitation” [Mesh]. The search was conducted in Google Scholar, PubMed, and Scopus databases.

### 2.3 Selection of Studies

A final selection of studies was made by two authors independently. All papers were reviewed primarily by titles and abstracts, excluding irrelevant articles. Also, if a full text was unavailable, study authors were contacted and asked to provide it. If no response was obtained, that study was discarded. Exclusion criteria included non-English/Persian articles without reliable translation, lack of full text after author contact, and studies not differentiating LAS from early post-hypoxic myoclonus.

The reviewers conducted a full review of each study’s text afterward. The reviewers generated BibTex files for the studies they considered eligible and then used a bibliographic manager to eliminate duplicates. Upon independent review by the two reviewers, each study was deemed eligible by consensus. Third reviewers made final decisions when no agreement was reached. This review was conducted following PRISMA 2020 guidelines; the detailed search strategy, inclusion/exclusion criteria, and PRISMA flow diagram are provided in Supplementary File 1.

## 3. Results

We collected 110 articles from databases such as Google Scholar, PubMed, and Scopus between 1970 and 2022 following the first screening phase of this study. Twelve non-English/non-Persian articles without reliable translation were excluded, and 15 were removed due to the inaccessibility of their full text, following a detailed evaluation of the studies.

In total, 83 articles were selected for review. These were primarily case reports, which were reviewed extensively to understand Lance-Adams syndrome’s pathophysiology, imaging findings, treatments, and prognosis. By analyzing these articles, we gained insight into the current state of knowledge about this condition and the methods used for its diagnosis, management, and prognosis. Lance-Adams syndrome and its underlying pathophysiology, imaging findings, the effectiveness of different treatment options, and prognostic indicators have been clarified by this review.

## 4. Discussion

### 4.1 Pathophysiology

LAS is characterized by myoclonus, a symptom that can occur within hours, days, or weeks of a hypoxic event. The disorder has been suggested to have both subcortical and cortical origins [7]. A study by Guo et al. [8] reported two cases of LAS following cardiopulmonary resuscitation (CPR). One of the cases had been triggered by emotional excitement of a cortical origin.

Neurotransmitters such as serotonin and gamma-aminobutyric acid (GABA) may contribute to LAS. There is evidence that serotonin loss has been linked to post-hypoxic myoclonus, and GABA is thought to suppress the effects of serotonin [9].

Hypoxic myoclonus can result from several causes, including cardiac arrest, respiratory obstruction, anesthesia accidents, carbon monoxide poisoning, hemorrhagic shock, surgery, and hypoxia from hanging or drowning [10]. Several mechanisms have been identified in the brain that may lead to hypoxic myoclonus, including abnormal cortical discharges, abnormal circuits in the cerebellum, brain stem, and thalamus, and imbalances in neurotransmitters [9].

There is no clear understanding of the exact pathophysiology of LAS; however, imaging studies have shown that several areas are involved, including the pontine tegmentum, mesencephalon, ventrolateral thalamus [11], temporal lobe [4], basal ganglia [12], cerebellum [13], hippocampus, and frontal lobe [14] using imaging techniques such as magnetic resonance imaging (MRI), positron emission tomography (PET), and single-photon emission computed tomography (SPECT).

There is also evidence that repetitive firing of thalamocortical fibers initiated by the thalamic ventrolateral nucleus and the dentato-rubro-olivary circuit (Guillain-Mollaret triangle) may contribute to the development of palatal myoclonus. Additionally, myoclonus may occur due to lesions in the circuitry between the olivary nucleus, red nucleus, and deep cerebellar nuclei [15].

**Figure 1:**
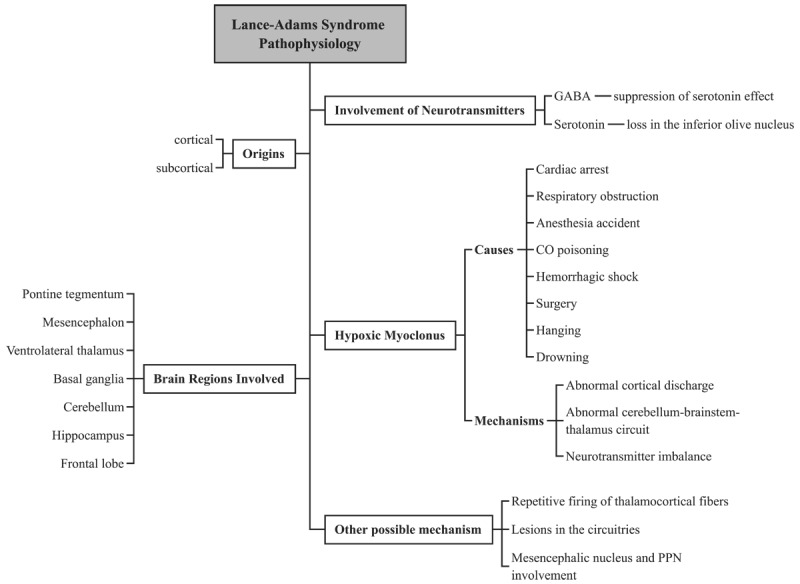
Lance-Adams syndrome pathophysiology. This figure illustrates Lance-Adams syndrome’s possible origins, neurotransmitter involvement, and probable causes and mechanisms underlying hypoxic myoclonus, which may affect various brain regions, consistent with prior imaging and metabolic studies [11,14]. *Abbreviations: GABA: gamma-aminobutyric acid. CO: carbon monoxide. PPN: pedunculopontine nucleus*.

A mechanism has been described by Song et al. that involves the mesencephalic nucleus and pedunculopontine nucleus (PPN), as well as the inferior olivary nucleus, cerebellum, and red nucleus from subcortical origin. This study demonstrated that a cerebellar lesion could disrupt the pathways linking the cerebellum to the thalamus and subthalamic nucleus, resulting in myoclonus caused by PPN pathology and preventing patients from walking independently for six months [15]. The LAS pathophysiology is summarized in Figure 1.

### 4.2 Diagnosis

**Figure 2:**
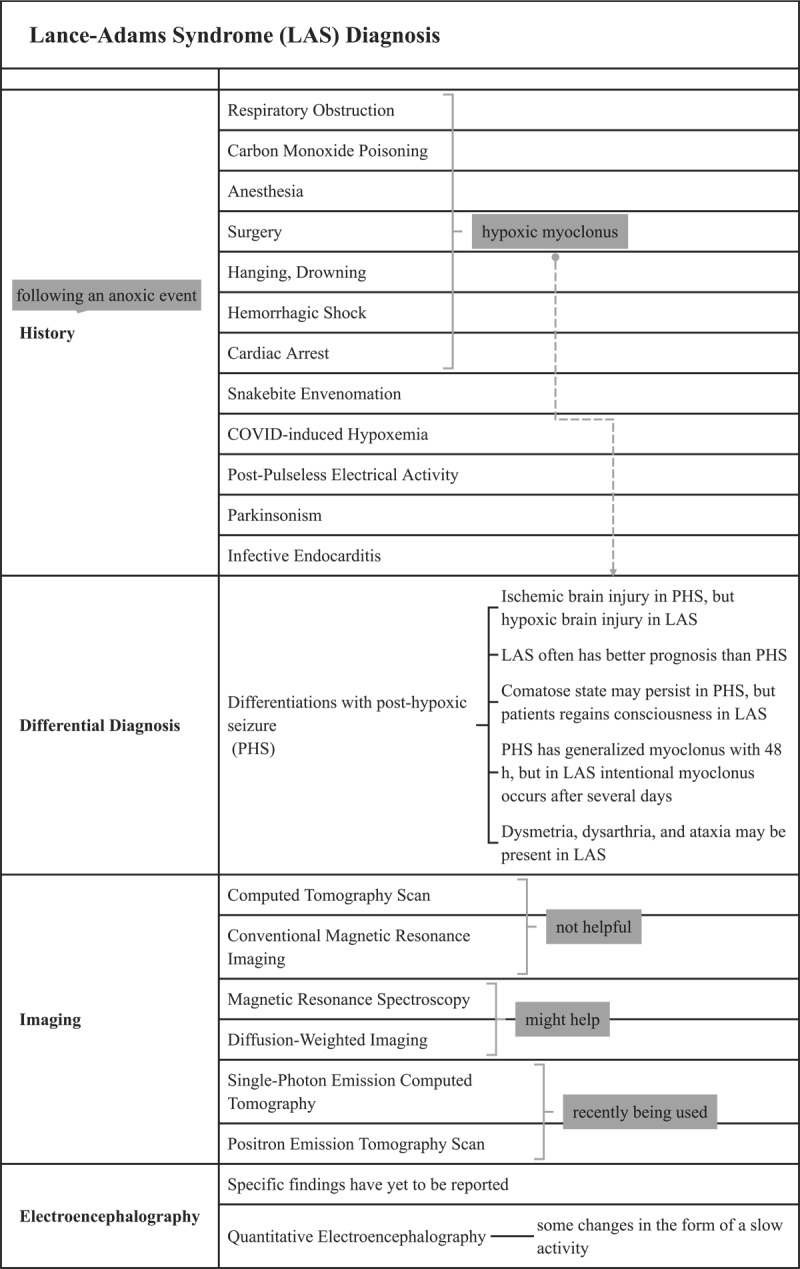
Lance-Adams syndrome diagnosis. This figure presents different aspects of the diagnosis of Lance-Adams syndrome. An in-depth patient history should be obtained, as this condition usually occurs following hypoxia, and a differential diagnosis should be considered. The use of some imaging modalities may assist in the diagnosis. Even though no specific EEG findings have been reported, quantitative EEG may reveal some changes. *Abbreviations: LAS: Lance-Adams syndrome. PHS: post-hypoxic seizure. EEG: electroencephalography*.

As shown in Figure 2, LAS is diagnosed primarily based on the patient’s medical history and symptoms, with recovery from an anoxic event serving as the primary diagnostic criteria [3]. In addition to cardiac arrest [14,16,17,18,19,20,21], other causes have been reported, including snakebite envenomation [22], COVID-19-induced hypoxemia [7,23], infectious endocarditis [24], post-pulseless electrical activity [25], parkinsonism [26], near drowning [27], and strangulation [28] as well. A second cardiac arrest has been observed in some patients with LAS who did not exhibit myoclonus after the return of spontaneous circulation [17].

The LAS is characterized by generalized action myoclonus following cardiac arrest and coma, usually accompanied by hypoxia [29]. There are also other associated features, such as dysmetria, dysarthria, and ataxia, with relative preservation of cognitive function. It is important to note that this syndrome has the potential to become chronic and that long-term antiepileptic treatment may be necessary [30].

#### 4.2.1 Differential Diagnosis

Myoclonus occurs in about 20% of cardiac arrest survivors [31,32]. Post-hypoxic seizures (PHS), or myoclonic status, can complicate the differential diagnosis of LAS. Post-hypoxic seizures differ from LAS because the symptoms persist after regaining consciousness. It is possible for a patient to remain comatose in PHS; however, in LAS, the patient can regain consciousness. Typically, myoclonus appears within 48 hours of CPR in PHS, while intentional myoclonus may appear several days following the hypoxic brain injury [3].

It should also be noted that status myoclonus is distinguished from LAS because it is associated with a poor prognosis and is characterized by clinical and electroencephalographic differences [31,33,34,35]. Early myoclonus patients show suppression-burst electroencephalogram (EEG) patterns associated with high-amplitude, diffuse polyspikes, which are associated with a dismal prognosis (“status myoclonus”). Furthermore, LAS can be associated with a continuous background characterized by narrow, midline-centered spike waves [35].

In addition, myoclonic status is thought to be caused by an ischemic brain injury with neuronal necrosis. While it has been reported that in LAS, hypoxic brain injury without irreversible infarction may be the case [36].

#### 4.2.2 Imaging

Anatomical and pathophysiological bases for LAS have been demonstrated using advanced neuroimaging techniques such as brain SPECT and PET. Using these techniques makes it possible to determine the metabolism of the affected areas in LAS. Compared to a control group, Frucht et al. found that the LAS group had significantly higher glucose metabolism in the pontine tegmentum, mesencephalon, and ventrolateral thalamus [11]. In another study on patients with LAS, it has been reported that there is mild hypoperfusion in the left temporal lobe in one patient, as confirmed by brain SPECT, and a mild bilateral decrease of glucose metabolism in the frontal lobes in another patient, as confirmed by brain PET. A study by Lee et al. also confirmed these findings. According to two studies, SPECT showed reduced blood flow in the right basal ganglia and left temporal lobe of patients with LAS. As opposed to this, cerebral blood flow was increased on the brain’s frontal, temporal, and parietal surfaces [4,14].

In the diagnosis of LAS, brain computed tomography (CT) or MRI is not helpful [6]. Some studies have examined changes in the brain after LAS occurrence using MRI. Some suggested that in conventional MRIs, diffuse atrophy may be visible [37,38]. An MRI was performed on patients 2.5 years after LAS in a study by Werhahn et al. Four patients in this study showed no abnormal changes, four displayed non-specific abnormalities, such as mild cerebral atrophy and cerebellar atrophy, and four had hemispheric or cerebellar infarctions [39]. As a result of analyzing MRI results, Ferrlazzo et al. reported transient thalamic and cerebellar involvement without permanent damage to the brain architecture in LAS patients, possibly due to reversible cytotoxic edema [6].

An advanced MRI technique used in surveying the brain is magnetic resonance spectroscopy (MRS). According to Richardson et al., the N-acetyl aspartate to creatine ratio (NAA/Cr ratio) was significantly decreased in the normal posterior cingulate gyrus (PCG) cortex and parietal white matter regions, indicating hypoxic brain damage. This is also a reasonable description of the EEG findings in the LAS [40]. A woman with cardiorespiratory arrest was also reported to have a decreased NAA peak in both hippocampi [14].

The diffusion-weighted imaging (DWI) sequence has also been used for LAS. According to Zhang et al., no abnormal findings were observed in two LAS patients who had undergone conventional MRI and DWI examinations in the early stages of the disease, respectively, three weeks and nine months following cardiorespiratory arrest [14]. A DWI study conducted over 24 hours of post-anoxic coma demonstrated basal ganglia, cerebellar, or cerebral cortex involvement in most patients. T2-weighted images, on the other hand, were normal or revealed mild hyperintensities of the basal ganglia [41].

#### 4.2.3 Electroencephalogram

An electrophysiological study can be used to identify cortical myoclonus before consciousness returns in the event of LAS [42]. On EEGs of LAS patients, multiple sources and multifocal lesions are commonly seen, with approximately half exhibiting epileptic discharges [43]. A literature review revealed that burst suppression was the most commonly reported EEG pattern, whereas alpha comas were not expected [38]. Several previously reported LAS cases had shown a decrease in EEG frequency or spike waves. Myoclonic jerks may frequently, but not always, be associated with multiple spikes followed by slow waves on the EEG [6,38].

Quantitative EEG (QEEG) is a newly used modality to examine LAS, with changes in the form of slow activity observed during extended follow-up of LAS patients in a study by Szczepańska et al. [44]. However, specific and characteristic diagnostic findings of EEG in LAS patients have yet to be reported [4,45]. Representative EEG and imaging findings have been described in prior literature, although supplementary figures or videos could not be included in this review due to limitations.

### 4.3 Treatment

Since the etiology of LAS is unknown, treatment options are limited, and results are suboptimal. In the current state of the art, the primary treatment approach for LAS is a combination therapy that includes antiepileptic drugs such as clonazepam, sodium valproate, piracetam, and levetiracetam, which have been recommended as first-line treatments by Zhang et al. [14] and Polesin et al. [46]. The study by Frucht and Fahn [47] on 100 patients with LAS found that clonazepam, valproate, and piracetam were effective in treating 50% of the cases. In contrast, Lee et al. [4] concluded that clonazepam and levetiracetam effectively treat myoclonus. Myoclonus of the cortical region can be treated effectively with levetiracetam [48].

It has recently been suggested that perampanel (PER) might be used to treat LAS [49,50,51,52,53,54]. As a result of its selective inhibition of the 5-methyl-4-isoxazole propionic acid (AMPA) receptor can reduce excessive nerve cell excitation and Ca2+ inflow [50,55,56]. PER is shown to reduce seizures and myoclonus in a dose-dependent manner [57] and has a possible threshold dosage of 6 mg/day, with a maximum dosage of 12 mg/day, to avoid severe side effects, such as irritability, anxiety, violence, hallucinations, and weight gain [58]. To avoid side effects, the drug should be titrated slowly [59].

Sodium oxybate [60,61], cannabidiol [62], volatile anesthetic agents [63], levetiracetam [64,65], gamma-hydroxybutyrate [66], L-5-hydroxytryptophan [67], piracetam [68], levodopa [69], intrathecal baclofen [70], brivaracetam [71], lacosamide [72], and valproic acid [73,74,75] are among the treatments mentioned in the literature for LAS.

**Figure 3:**
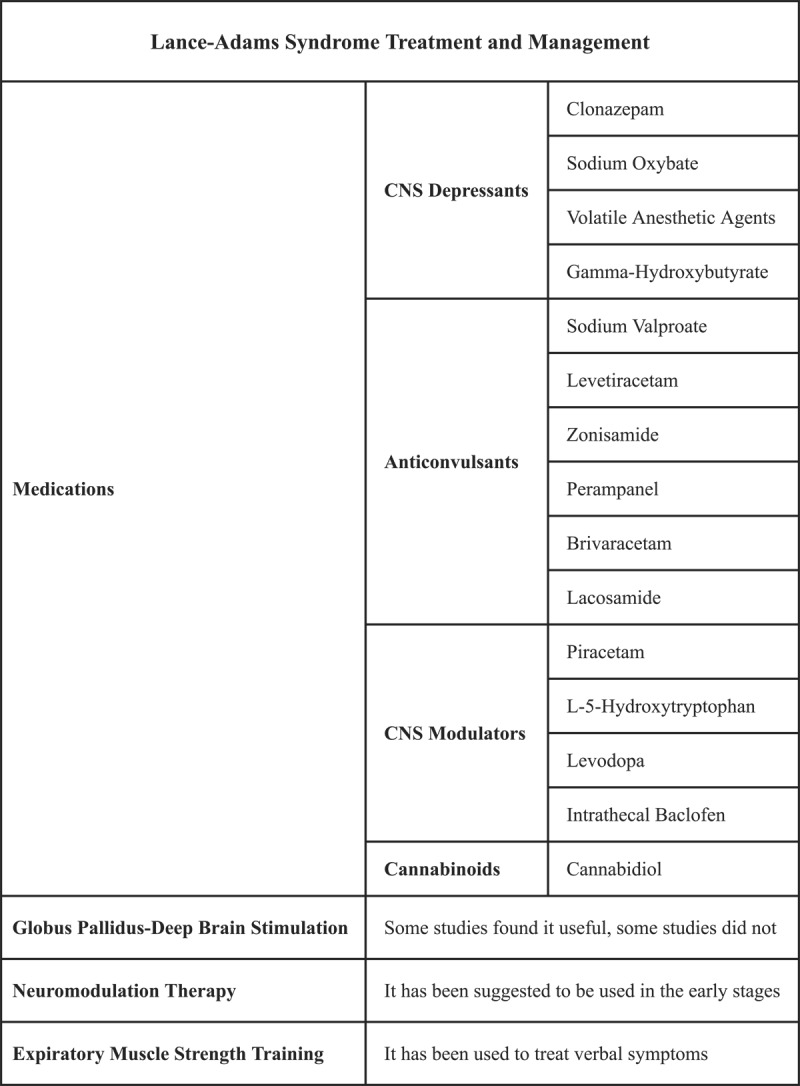
Lance-Adams syndrome treatment and management. This figure demonstrates possible treatments and management options for Lance-Adams syndrome. Various medications have been reported as having been used for the syndrome in past literature, including CNS depressants, anticonvulsants, CND modulators, and cannabinoids. It is controversial whether globus pallidus-deep brain stimulation is beneficial. Moreover, neuromodulation therapy has been suggested to be used in the early stages of the syndrome, as well as expiratory muscle strength training to alleviate verbal symptoms of the disease [49,50,51,52,53,54].

Another treatment method of LAS is stimulating the globus pallidus with deep brain stimulation (GPi-DSB). Some recent studies have suggested that GPi is a preferred treatment option in myoclonus patients, and this technique has been successfully employed in previously published LAS cases [12,55,76,77,78]. Despite this, the prolonged outcome of this method has yet to be well studied. During the three-year follow-up period following GPi-DBS 14 years after the injury, Ozturk et al. did not demonstrate a significant improvement in action or resting myoclonus. Additionally, it has been suggested that neuromodulation therapy would be more effective in the early stages of the disease [79]. It has also been shown that expiratory muscle strength training (EMST) can be practical for treating verbal symptoms associated with LAS [80]. All data regarding LAS treatment and management is presented in Figure 3.

### 4.4 Prognosis

Compared with early post-anoxic status myoclonus, LAS generally carries a more favorable functional prognosis; however, it often remains chronic and functionally disabling without individualized treatment [3,39], early diagnosis and treatment are essential to improving patients’ quality of life and minimizing their disabilities [39]. However, if LAS is not diagnosed on time and inappropriate anticonvulsant therapy is prescribed, the prognosis may be adversely affected. So, LAS should be considered if the patient exhibits uncontrolled myoclonus that conventional anticonvulsants cannot control after CPR has been performed and the patient has regained consciousness [4].

### 4.5 Limitations

This review is limited by the predominance of case reports and small series, which provide heterogeneous and often incomplete data. Demographic information such as age, sex distribution, and long-term outcomes was inconsistently reported and therefore could not be synthesized quantitatively. Furthermore, non-English and non-Persian articles without reliable translation were excluded, which may have led to missing relevant cases. These limitations precluded the performance of a meta-analysis and restricted our ability to provide aggregate descriptive statistics.

## 5. Conclusion

Lance-Adams syndrome is a rare form of myoclonus in which involuntary movements occur after a hypoxic event, such as a cardiac arrest. As this was the only recent review on this topic, this research was conducted to gain a comprehensive understanding of this syndrome. As a result of the review, the authors concluded that LAS has both subcortical and cortical origins and that neurotransmitters such as serotonin and GABA may play a role. Numerous brain areas may be involved in LAS, as evidenced by imaging studies. Although pharmacological intervention is the most common, non-pharmacological treatments have also been used with varying degrees of success, including deep brain stimulation and repetitive transcranial magnetic stimulation.

LAS is a chronic condition that can impact patients’ quality of life, and while treatment can help manage symptoms, patients may still have difficulties coping with the condition. However, the exact pathophysiology of LAS needs to be fully understood, and further research is necessary. Clinicians should consider early recognition and tailored pharmacologic therapy, while research should focus on standardized outcome measures and multicenter registries.

## Additional File

The additional file for this article can be found as follows:

10.5334/tohm.1074.s1 Supplementary File 1. PRISMA 2009 Flow Diagram.

## Declarations

This manuscript contains all required subheadings and information per journal instructions. The authors confirm the manuscript complies with all instructions to authors. Authorship requirements have been met and the final manuscript was approved by all authors. This manuscript has not been published elsewhere and is not under consideration by another journal.
