# Supplementary material for: Lance-Adams Syndrome: An Updated Review of a Rare Post-Hypoxic Complication
Source: Tremor Other Hyperkinet Mov (N Y). 2025 Sep 24;15:45. doi: 10.5334/tohm.1074 (PMC12466330; doi:10.5334/tohm.1074)
Supplement: Supplementary File 1. — PRISMA 2009 Flow Diagram. [file tohm-15-1-1074-s1.pdf]

PRISMA 2009 Flow Diagram

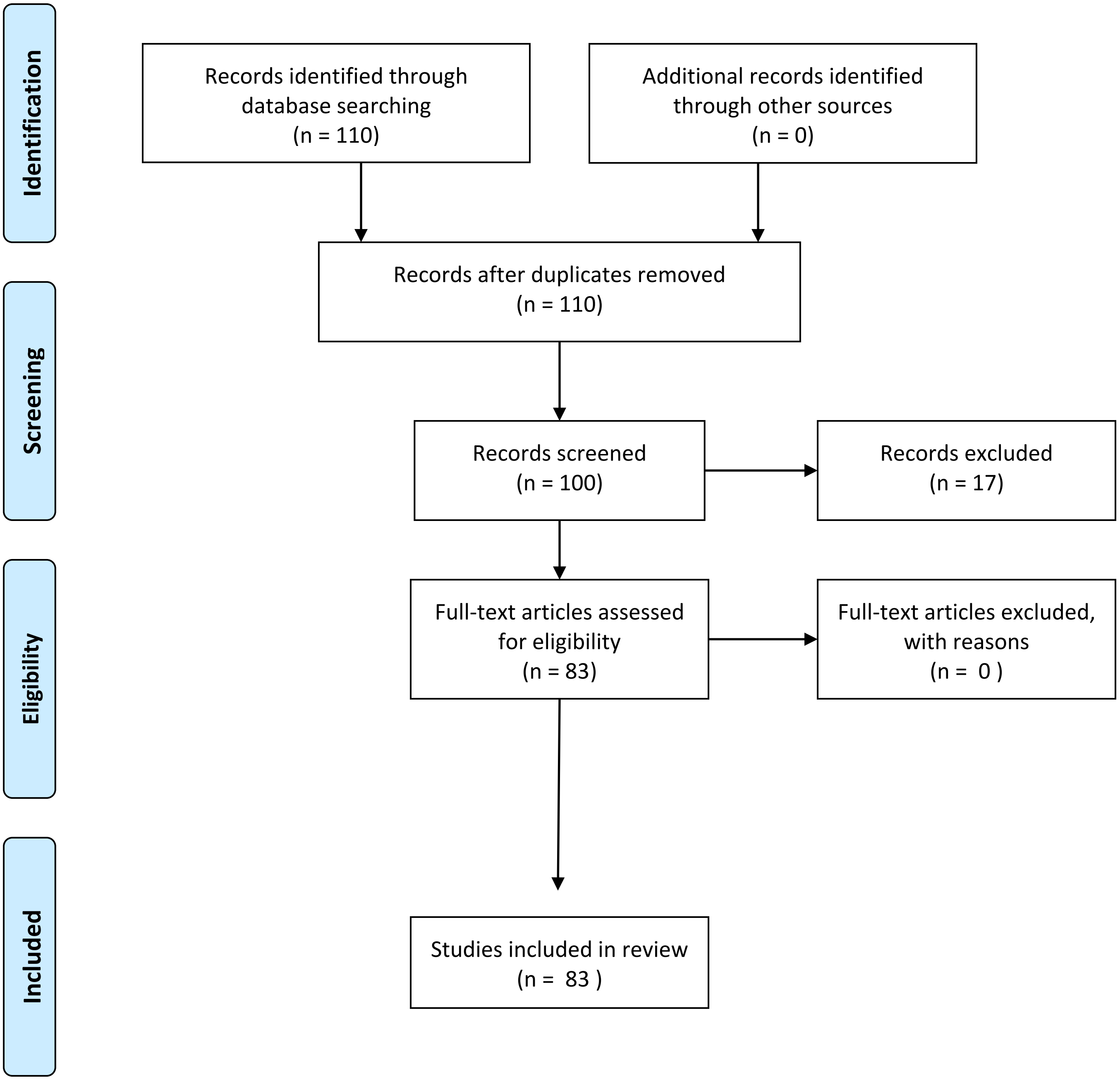

From: Moher D, Liberati A, Tetzlaff J, Altman DG, The PRISMA Group (2009). *P*referred *R*eporting *I*tems for *S*ystematic Reviews and *M*eta-*A*nalyses: The PRISMA Statement. PLoS Med 6(7): e1000097. doi:10.1371/journal.pmed1000097

For more information, visit www.prisma-statement.org.
